# Transvaginal Ultrasound Combined with Strain-Ratio Elastography for the Concomitant Diagnosis of Uterine Fibroids and Adenomyosis: A Pilot Study

**DOI:** 10.3390/jcm11133757

**Published:** 2022-06-28

**Authors:** Vlăduț Săsăran, Sabin Turdean, Claudiu Mărginean, Marius Gliga, Levente Ilyes, Ovidiu Grama, Lucian Pușcașiu

**Affiliations:** 1 Department of Obstetrics and Gynecology 2, Faculty of Medicine in English, George Emil Palade University of Medicine, Pharmacy, Sciences, and Technology of Târgu Mureș, Gheorghe Marinescu Street No. 38, 540136 Târgu Mureș, Romania; vlad_sasaran0403@yahoo.com; 2 Department of Morphopathology, Faculty of Medicine in English, George Emil Palade University of Medicine, Pharmacy, Sciences, and Technology of Târgu Mureș, Gheorghe Marinescu Street No. 38, 540136 Târgu Mureș, Romania; 3 Department of Obstetrics and Gynecology 2, Faculty of Medicine, George Emil Palade University of Medicine, Pharmacy, Sciences, and Technology of Târgu Mureș, Gheorghe Marinescu Street No. 38, 540136 Târgu Mureș, Romania; marginean.claudiu@gmail.com (C.M.); m11gliga@yahoo.com (M.G.); iliasz2001@yahoo.com (L.I.); ovi_grama@yahoo.com (O.G.); 4 Department of Obstetrics and Gynecology 1, Faculty of Medicine in English, George Emil Palade University of Medicine, Pharmacy, Sciences, and Technology of Târgu Mureș, Gheorghe Marinescu Street No. 38, 540136 Târgu Mureș, Romania; puscasiu@gmail.com

**Keywords:** uterine fibroid, adenomyosis, trans-vaginal ultrasonography, strain ratio elastography

## Abstract

Uterine fibroids (UFs) and adenomyosis (AM) represent two benign uterine conditions that can affect fertility and are most frequently commonly responsible for abnormal uterine bleeding and chronic pelvic pain. Their differential diagnosis still represents a challenge, and several authors advise the addition of elastography to transvaginal ultrasound (TVUS) for a more accurate imagistic recognition. Through this study, we aimed to assess the diagnostic accuracy of TVUS combined with strain-ratio elastography (SRE) in concomitant AM and UFs. We conducted a study on 17 patients diagnosed with concomitant UFs and AM undergoing hysterectomy and 46 healthy patients. TVUS combined with SRE was conducted in each patient, focusing on identifying rigidity patterns of the lesions. Significantly higher mean SR and maximum SR values were identified among both AM and UF lesions as opposed to controls (*p* < 0.01), with the highest tissue stiffness being encountered among AM lesions, which allows for the differentiation of UF (*p* < 0.01) and concomitant identification of both lesions. These results are reflected by higher cut-off values obtained for AM, both for mean SR (5.42 vs. 2.85) and maximum SR (5.80 vs. 3.30). TVUS combined with SRE showed good diagnostic performance in identifying coexisting UFs and AM within the same uterine specimen. Future studies on wider populations are required to validate our findings.

## 1. Introduction

Benign uterine conditions represent the most frequent gynecological pathologies affecting women of different ages, commonly during reproductive age [1]. These include uterine fibroids (UFs) and adenomyosis (AM), which constitute major etiologies of dysfunctional uterine bleeding, which often lead to anemia [2].

UFs, commonly known as uterine leiomyoma, are among the most common benign uterine tumors in women, which often cause dysmenorrhea, pelvic pain, menorrhagia, infertility and are sometimes completely asymptomatic [3]. Due to the diversity and vagueness of the symptoms, the true prevalence is not precisely known, but UFs are considered to affect up to 77% of all women, as microscopically identified on uterine specimens [4]. The incidence of UFs seems to be higher in black women, especially at younger ages, as opposed to white females [5]. Regardless of the age of diagnosis, individual or multiple UFs represent one of the most common indications for hysterectomy [2,3].

AM represents an abnormal growth of endometrial tissue within the myometrium, which causes inflammation and enlargement of the uterus due to hypertrophy of the endometrial glands. Most patients usually experience symptoms such as chronic pelvic pain, menorrhagia or dysmenorrhea, while only a few of them are completely asymptomatic [6]. Prevalence of adenomyosis is reported to be between 5 and 75%, with a mean of 27% in post-hysterectomy specimens [7,8,9]. Still, AM is frequently associated with other gynecological conditions [10]. Coexisting uterine disorders such as AM and UFs have been reported to be as high as 15–57%, and women presenting with coexistent pathologies are more likely to suffer from diffuse pelvic pain [11]. Studies conducted on women who underwent hysterectomy for UF and AM reported more frequent bothering dysfunctional symptoms (dysmenorrhea, dyspareunia, non-cyclic pelvic pain) compared to patients who underwent hysterectomy for UF only [12,13,14]. Thus, similarities in clinical presentation between the two disorders exist, but the intensity of symptoms increases in cases with concomitant AM and UF, and the probability of these two disorders coexisting maybe being underdiagnosed.

Transvaginal ultrasound (TVUS) is currently a first-line diagnostic tool because it is generally available, well-tolerated, cheap and relatively accurate when properly used [7,8,9]. MUSA (Morphological Uterus Sonographic Assessment) group created a universal reporting system of 2D TVUS ultrasound patterns for AM and UF, which aimed to improve diagnostic algorithms and reduce interobserver variability [11,15,16]. These features consist of uterine corpus evaluation (echogenicity and symmetry), detection of lesions of the myometrium and endometrium, as well as their patterns (location, size, number and shadowing) and assessment of Doppler color score of the myometrium (graded from 1 representing no color or minimal color, up to 4, which represents abundant color) [15]. Elastography represents a recent ultrasound imaging technology, commercially available, which can be split into two main systems: strain ratio elastography (SRE) and shear wave elastography (SWE) [17]. SRE is a qualitative method that involves the application of repetitive minimal pressure by the examinator on an anatomical structure, which provides information about region of interest (ROI) stiffness in comparison with surrounding tissue [17,18]. SWE, on the other hand, is a quantitative method that provides an estimative value of tissue stiffness expressed in kPa of ROI [17,19]. Both techniques are currently available, and their use in combination with TVUS is advised, as they are considered important supportive tools for the diagnosis of benign uterine disorders and their differentiation from malignant lesions [19,20,21]. Thus, due to its capacity to provide information about normal uterine tissue and other uterine lesions, elastography in combination with TVUS may constitute important assets in preoperative planning and differential diagnosis of uterine pathologies.

This study aims to evaluate the accuracy of TVUS combined with SE in detecting concomitant AM and UF within the same uterine specimen.

## 2. Materials and Methods

A prospective pilot study was conducted on 146 patients who presented to a tertiary Obstetrics and Gynecology Clinic in Romania over a timespan of two and a half years (May 2019–November 2021).

### 2.1. Study Population and Division of Study Groups

This study enrolled 100 patients who underwent total hysterectomies for suspicions of AM, UF or coexisting AM and UF, whose diagnosis was established based on 2D TVUS in combination with SRE. Furthermore, during the same timespan, we enrolled 46 healthy women of reproductive age who presented for regular check-ups and constituted the control group. Patients included in the study group complained of symptoms such as menometrorrhagia (especially treatment-resistant), dysmenorrhea, chronic pelvic pain or symptoms associated with pelvic compression caused by the increased uterine volume. Furthermore, each of the patients enrolled in the study in whom the suspicion of a benign uterine pathology was raised was of reproductive age and due to undergo a total hysterectomy. Exclusion criteria were represented by personal history of malignancy, ongoing pregnancy, previous or present exposure to exogenous hormones (Gn-Rh agonist use, administration of oral contraceptives, hormonal intrauterine device-IUD implantation), or concomitant diagnosis of a uterine infection. Histopathological examination constituted the reference diagnostic method, and patients with a sole diagnosis of UF, AM or concomitant malignancy were ruled out of the study. Thus, we expected a limited number of patients among the study group, as data provided by multicentric studies have shown that a concomitant diagnosis of AM and UFs is encountered in no more than 20–28% of uterine specimens after total hysterectomy [22,23].

### 2.2. Ethics

This research complied with the principles stated in the Declaration of Helsinki. Every subject approved to enrollment in the study by signing an informed consent form that contained a comprehensive description of the study protocol prior to inclusion. The research protocol was approved by the Ethics Committee of the Clinical County Hospital Mures and the one of the Ethics Committee of “George Emil Palade” University of Medicine, Pharmacy, Science and Technology of Targu Mures (numbers 4916/2019 and 1205/2020).

### 2.3. Ultrasound Examination

**Figure 1:**
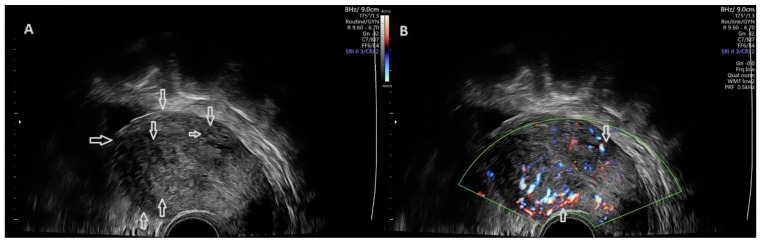
(**A**) B–mode, TVUS characteristics of uterine adenomyosis (featured in the superior part of the image) and a uterine fibroid (inferior part of the image) (pointed out through the arrows): globally increased uterine volume with myometrial cysts and ill-defined lesion; round lesion, with clear, distinct borders and fan-shaped shadowing. (**B**) B–mode TVUS with Power Doppler mode features of uterine adenomyosis (superior part of the image) and uterine fibroid (inferior part of the image) (highlighted by the arrows): trans–lesional blood flow; circumferential blood flow.

Patients from the study group and the healthy group were scheduled for TVUS combined with SRE. Images were acquired and evaluated by a single trained ultrasonographist. For ultrasound examination and strain ratio elastography, we used Voluson E10 BT16, Voluson E8 BT18 and Voluson E10 BT20 ultrasound machines (General Electric Healthcare, Chicago, IL, USA) with RIC-5-9-D 9 MHz vaginal probes. A 2D transvaginal ultrasound examination of the uterus was initially performed in both groups for the identification of any abnormality (Figure 1).

In patients with suspicion of AM and UF, MUSA criteria were used for the diagnosis of the lesions [15]. Due to the fact that apparently healthy endometrial tissue was used as a referral for SRE, patients were called-up for ultrasound examination between the 8th and 14th day of the menstrual cycle. In B-mode ultrasonography, uterine fibroids were described as well-defined, round lesions localized inside the myometrium or in its neighboring tissue. Shadows were identified at the periphery or inside the lesion (fan-shaped shadowing). Other characteristics of uterine fibroids were circumferential blood flow, symmetry, heterogeneity and hypoechoic/hyperechoic masses. Adenomyosis was ultrasonographically described as a poorly-defined lesion, echogenic and/or cystic striations. A globally enlarged uterus was usually associated, whereas myometrial cysts, translesional blood flow and an interrupted junctional zone were also commonly encountered features. Fan-shaped shadowing was a common feature of both uterine fibroids and adenomyosis [15].

### 2.4. Strain Ratio Elastography Evaluation

**Figure 2:**
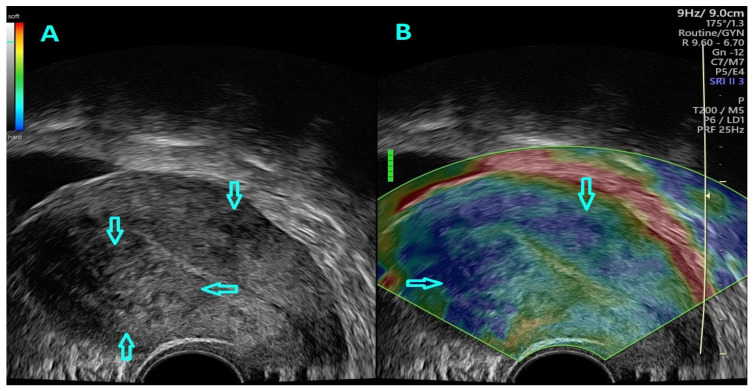
(**A**) B–mode, TVUS characteristics of uterine adenomyosis (superior part of the image) and a uterine fibroid (inferior part of the image) (pointed out through the arrows) (**B**) SRE evaluation of an image acquired through dual-mode, real–time TVUS which depicts coexisting adenomyosis and a uterine fibroid.

Strain ratio elastography was performed in real-time using a split-screen mode and was preceded by a 2D TVUS of the uterus (Figure 2).

**Figure 3:**
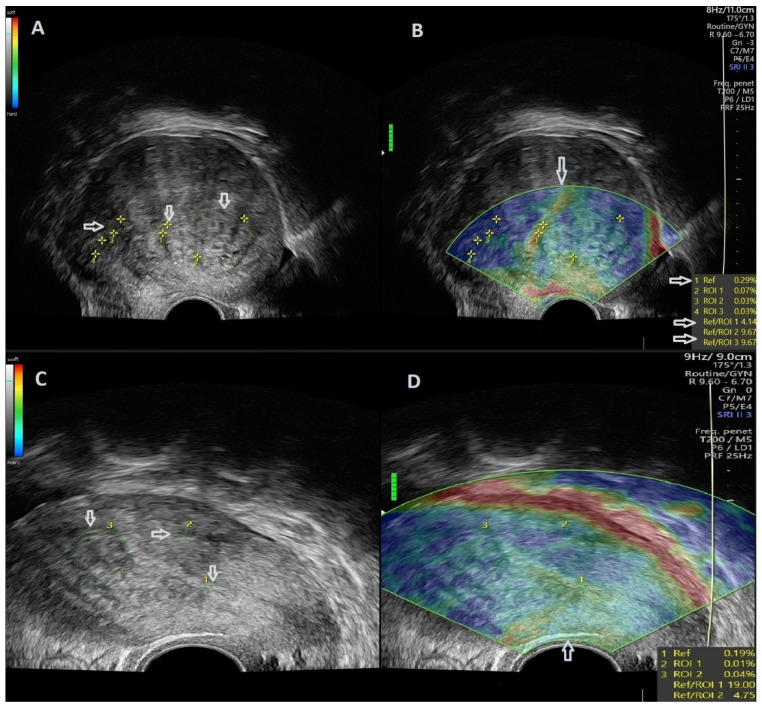
(**A**) Arrows depict ROI placement for comparative assessment of healthy, referral tissue with adenomyotic lesions (left side of the image) and a uterine fibroid (right side of the image). (**B**) SRE evaluation of an image acquired through dual-mode, real-time TVUS illustrating a uterine fibroid and adenomyosis. (**C**) Arrows depict ROI placement for comparative assessment of healthy, referral tissue and adenomyotic with adenomyotic lesions (superior part of the image) and uterine fibroid tissue (inferior part of the image). (**D**) SRE evaluation of an image acquired through dual-mode, real-time TVUS illustrating a uterine fibroid and adenomyosis.

This examination was performed for each group of patients, the study group, and the control group as well. Thus, the elastogram was viewed in real-time dual-mode in parallel with the ultrasonographic 2D image. In order to obtain the elastosonographic images, external pressure was applied using the vaginal probe, through which deformation and compression of the targeted tissue were ensured, obtaining a strain ratio value. The pressure was exerted by the examinator in accordance with the quality indicator of the ultrasound machine, thereby indicating the degree of pressure required to obtain the maximum quality of the images. Therefore, using this indicator as a reference, we were able to obtain reproducible values in iterative measurements. We performed three cycles of gentle compression and relaxation. The encoding of the elastosonographic image obtained was realized using 4 colors, namely yellow, green, red and blue. Thus, both red and yellow colors were representative of soft tissue, blue color represented rigid tissue, whereas the green color was attributed to the tissue with an intermediate degree of stiffness. The stiffness of the studied tissues was compared to the one of the adjacent endometria. Therefore, the color map and strain ratio values obtained were indicators of the rigidity of the studied tissues. The first ROI is represented by the reference tissue (the adjacent endometrium), and the others are represented by the target tissue (Figure 3).

ROIs were settled between 10 mm and 80 mm. Each lesion was measured three times, similar values were obtained, and their average was afterward calculated. Thus, mean as well as maximum strain ratio values (mean SR and max SR) were obtained.

### 2.5. Statistical Analysis

Quantitative variables (age, BMI, mean and max SR) were first assessed with the help of descriptive statistics and represented as mean ± standard deviation (SD). A normality test (Shapiro–Wilk test) evaluated the distribution of these variables and to decide the appropriate mean comparison test. Therefore, mean comparison was performed using the *t* test with Welch’s correction for variables complying with a Gaussian distribution, whereas Mann–Whitney test was applied for non-Gaussian distributed variables. In the case of comparison of more than two data sets, Kruskal–Wallis non-parametric tests were used. For comparison of binary data (curettage history or C-section history), Chi square test was used, with the consequent calculation of odds ratio (OR). Receiver operator characteristics (ROC) analysis was performed for mean SR and maximum (max) SR values of both adenomyosis and leiomyoma lesions. Area under the curve (AUC) values were represented graphically, and cut-off values were obtained, with adjacent sensitivity and specificity, for both mean and max SR. The significance threshold was defined as *p* < 0.05, in accordance with a confidence interval (IC) of 95%. GraphPad Prism 9.0.2 software was used for statistical analysis.

## 3. Results

After excluding patients with isolated UFs and AM and one case of concomitant cervical cancer, the study population consisted of 63 patients. Out of these, 17 were histologically diagnosed with concomitant adenomyosis and leiomyoma and represented the study group, whereas controls accounted for 46 patients without any abnormal ultrasonographical features of the uterus and ovaries. The identification of benign uterine lesions was based upon MUSA criteria, identified during TVUS, as previously detailed in the materials and methods section.

Table 1 details a comparison of age, BMI and personal history of common surgical interventions in young women between the two study groups. A significantly higher mean age can be noted within the study group (47.12 ± 4.10 versus 37.8 ± 7.76, *p* < 0.01). Moreover, a positive history of curettage was positively associated (*p* = 0.01) with coexisting adenomyosis and leiomyoma, with an OR of 4.04 (CI: 1.20–12.97). Insignificant differences between the two groups were noted in terms of BMI (*p* = 0.39) or history of C-section (*p* = 0.48).

**Figure 4:**
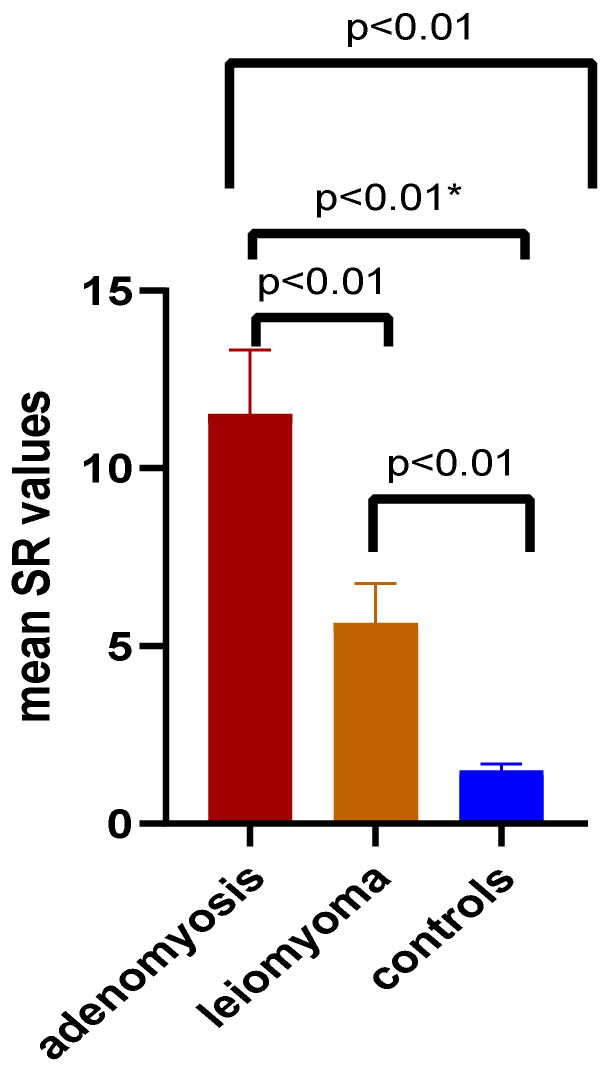
Comparison of mean SR values between adenomyotic, leiomyoma lesions and controls. Legend: SR—strain ratio; *—Mann–Whitney test was used (*t* test with Welch’s correction was used for other comparisons of two data sets).

**Figure 5:**
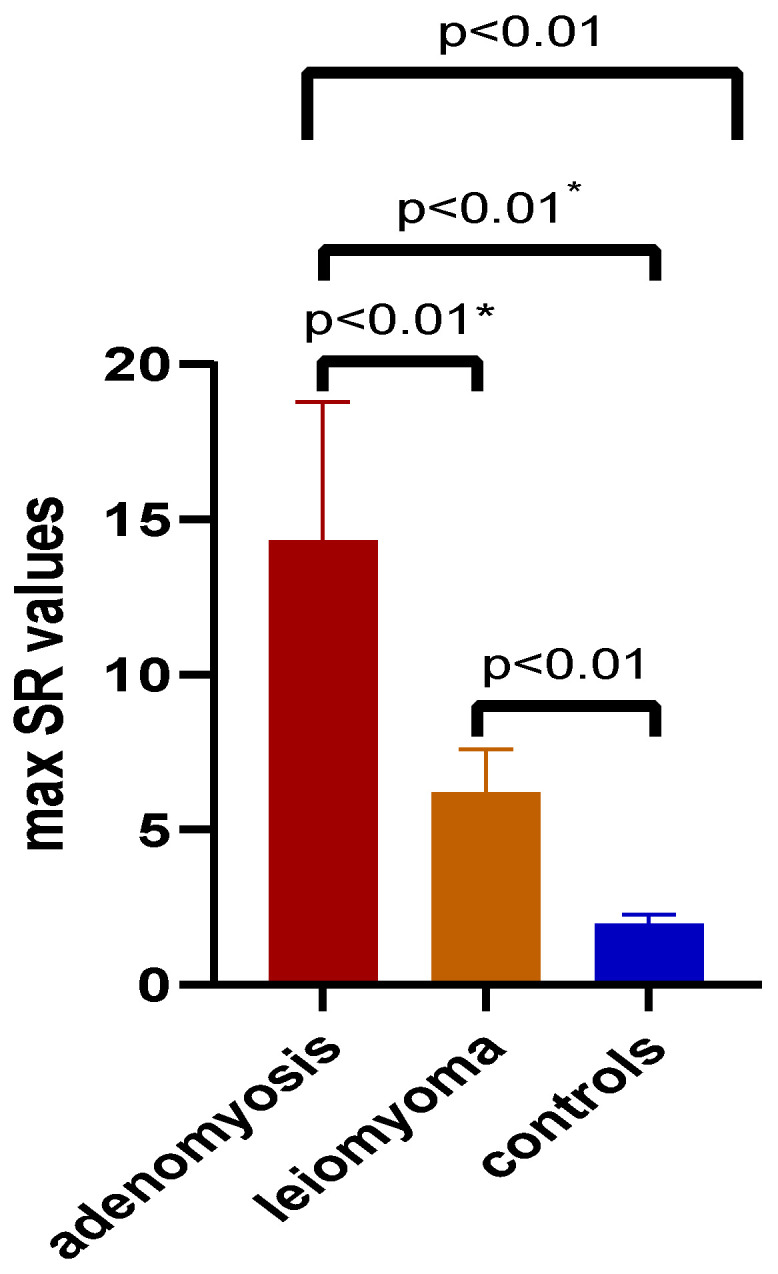
Comparison of max SR values between adenomyotic, leiomyoma lesions and controls. Legend: SR—strain ratio; *—Mann–Whitney test was used (*t* test with Welch’s correction was used for other comparisons of two data sets).

Mean SR and max SR values were obtained for both adenomyosis and leiomyoma lesions, as well as for normal uterine tissue, through shear-wave elastography. The comparisons of mean SR between adenomyosis, leiomyoma and controls are depicted in Figure 4. A significant increase (*p* < 0.01) was seen in mean SR values in both adenomyosis (11.52 ± 1.80 SD) and leiomyoma (5.63 ± 1.14 SD) lesions as opposed to controls (1.49 ± 0.18 SD). The highest mean SR values were identified among the adenomyotic lesions, the discrepancy being obvious not only in comparison to controls but also in comparison to leiomyoma (*p* < 0.01). Similar results were obtained with comparison of max SR values, as illustrated in Figure 5. The highest max SR values were identified among adenomyosis lesions (14.29 ± 4.49 SD), with important differences from leiomyoma (6.17 ± 1.41 SD) or controls (1.94 ± 0.30 SD).

Elastography findings among the study group were compared with histological results. A microscopic diagnosis of coexisting adenomyosis and leiomyoma was established in 17 patients, but this diagnosis was initially suspected in only 13 patients. Two cases had been initially diagnosed with isolated adenomyosis, whereas in another two cases, a diagnosis of leiomyoma was initially suspected based on ultrasound and elastography features.

**Figure 6:**
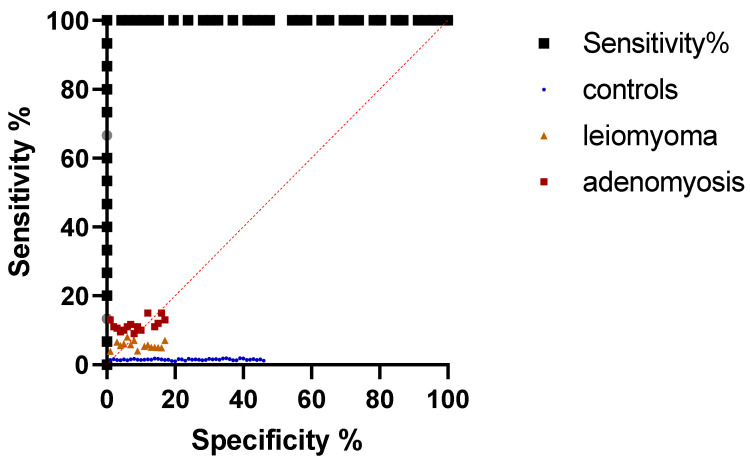
ROC curve of mean SR values for differential diagnosis of adenomyosis and leiomyoma from healthy uterine tissue.

**Figure 7:**
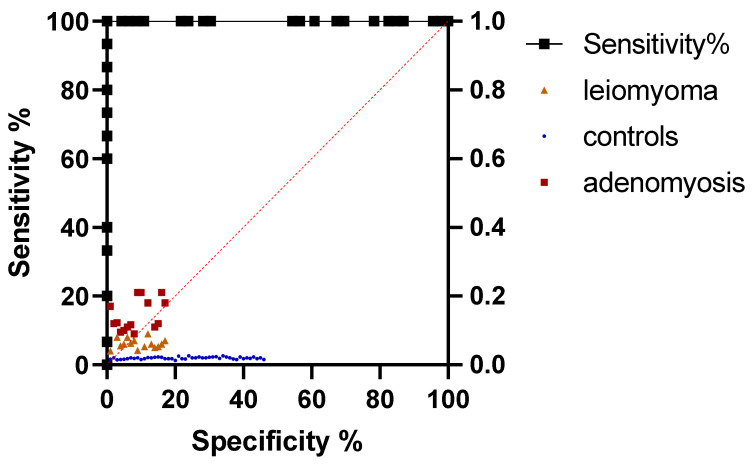
ROC curve of max SR values for differential diagnosis of adenomyosis and leiomyoma from healthy uterine tissue.

In the case of mean SR, after values of AUC of 1.00 for both adenomyoma and leiomyoma were obtained (*p* < 0.001). In the case of adenomyosis, a cut-off value of 5.42 was identified for mean SR (with a sensitivity and specificity of 100%). In the case of leiomyoma, a cut-off value of 2.85 was of the same sensitivity and specificity. For max SR, sensitivity and specificity of 100% were obtained for cut-off values of 5.80 for adenomyosis and 3.30 for leiomyoma. Values of AUC of 1.00 were also obtained for both max SR values. These results are depicted in Table 2 and Table 3. ROC curves for mean SR and max SR are illustrated in Figure 6 and Figure 7.

## 4. Discussion

Ultrasound elastography, commonly entitled sonoelastography, is a combination of non-invasive methods used as an increasingly diagnostic valuable tool in several pathologies, such as breast tumors, thyroid nodules, lung lesions, chronic liver disease and chronic kidney disease [24,25,26,27,28]. TVUS in combination with elastography is successfully used in gynecological practice to identify various uterine pathologies, both malignant and benign [17,21,29]. In contrast to other non-invasive diagnostic methods such as CT or MRI, this method is much more accessible and allows a correlation between clinical and imagistic examination in real time [7,30,31]. This method poses a great advantage over CT examination by not exposing the patient to radiation and being a quickly available, reproducible examination whenever needed [32]. Furthermore, this imagistic investigation requires a shorter examination time than MRI and involves lower costs [4,28]. Elastography has proven its effectiveness in diagnosing uterine fibroids in the case of both SWE and SRE [7,19]. According to literature data, SRE might be a valuable tool in the diagnosis of adenomyosis, but the appliance of SWE revealed contradictory results [9,18,19,33]. On the other hand, in the differential diagnosis of concomitantly existent uterine fibroids and adenomyosis, only SRE has proven its effectiveness so far [9,16,18].

Through this study, we tried to observe the use of transvaginal ultrasound and elastography in the diagnosis of benign uterine pathologies and to evaluate the ability of this method to differentiate these pathologies in the case of their coexistence [6,9]. Imagistic results were compared to histopathological findings (the gold standard diagnostic method). For a depiction of SR values, we compared the supposedly healthy endometrial tissue with the studied tissue (UF, AM or normal myometrium), in contrast to other authors, which used surrounding myometrial tissue as reference [7,9,19]. This approach was chosen due to the high probability of the surrounding myometrial tissue suffering changes in elasticity in the case of pathological uterine species, which can influence the measurement results, according to Liu et al. [18].

In our study, min and max SR values were obtained, with adenomyotic tissue being the stiffest, followed by fibromatous tissue and normal myometrium, these results being similar to the ones depicted by Liu et al. and within one of our previous studies, conducted on patients with AM or UF [16,18]. Still, Liu et al. reported lower numerical values, which may be explained by the differences between the manufacturers of the ultrasonography devices used [30]. Contradictory results compared to our study were obtained by Frank et al. and Görgülü et al.; both of these studies identified a lower rigidity of the adenomyotic tissue than the one of the fibromatous tissue [7,9,30]. However, both studies used different methods to obtain reference ratio values by comparing targeted tissue to the supposedly healthy adjacent myometrial tissue; this approach explains the differences from our findings [7,9,31].

The study group and the control group were selected respecting certain exclusion criteria, such as the previous lack of use of hormonal medication or absence of concomitant or previous malignant pathologies. Moreover, the study included only patients of reproductive age. These study population selection criteria were also found in other studies and are justified by the influence of hormonal therapy on uterine tissue stiffness, as well as by the miscellaneous findings of SR values in patients with malignant conditions [7,18]. Moreover, post-menopause can influence the stiffness of uterine tissue, as demonstrated by Frank et al. [9]. Our study population adhered to the aforementioned criteria, but we obtained a significant difference in terms of age between the study group and controls. This can be explained by the fact that controls were required not to have any type of ongoing or previous uterine disorder and no personal history of contraceptive use, and women complying with these criteria are obviously younger than the ones diagnosed with benign uterine disorders who present symptoms usually at older ages. Similar age differences were reported by Frank et al. in their study [9].

The accuracy of TVUS in the combined diagnosis of uterine fibromatosis and adenomyosis seems to be rather low due to the fact that UFs misshape uterine structure [34]. The combined use of sonoelastography might positively influence the correct diagnosis of benign uterine disorders; this technique is also being evaluated in comparison with other imaging techniques. A comparison with RMI revealed that TVUS with elastography seems to be superior in terms of sensitivity and specificity, but the authors of the study acknowledged that this conclusion should be certified by future studies on larger populations [30]. The superiority of TVUS combined with elastography is still debated by various factors, which can influence its accuracy. Among others, the examiner’s experience and level of training can be important elements that influence the correct diagnosis of benign uterine pathologies [8]. Although, in the case of uterine fibromatosis, the TVUS diagnosis can be easily reached, regardless of the examiner, an accurate ultrasound diagnosis of adenomyosis is highly dependent on the subjective perception of the examiner and his/her experience [31,34,35]. Hence, the combined diagnosis of these pathologies is mostly operator-dependent, and once the suspicion is raised by TVUS, elastography can provide an advantage in establishing the presence or absence of concomitant lesions of AM and UF [16,30,34]. The subjective perception of the examiner might have also influenced the inaccurate diagnosis of 4 patients included in our study, in which only one of the two pathologies was suspected.

Our study also tried to analyze some important risk factors involved in the appearance of adenomyosis and fibromatosis, given previous reports that sustained those interventions on the uterine cavity such as dilation and curettage may represent risk factors in the evolvement of these pathologies [13,22,36,37]. We also identified a significantly higher prevalence of positive curettage history among our study group. However, another study proved a lack of association between these interventions and AM or UF [38]. Cesarean section dd is not associated significantly with a diagnosis of AM and UF, in a similar manner to the results reported by Taran et al. [23].

The main limitations of this study are the small group of patients and its unicentric character. The low number of patients might be explained by the relatively rare ultrasound diagnosis of an association between these two pathologies and might represent the main risk for type II statistical errors [39]. In order to validate our findings, it would be necessary to expand the research on different types of populations from various geographical areas and on a larger number of patients.

Although our study enrolled a relatively limited number of patients, it has so far enrolled the widest population with a combined diagnosis of UF and AM who were assessed preoperatively with TVUS combined with sonoelastography. Furthermore, another strength was the use of histology as a diagnostic reference, which is still regarded as the gold standard diagnostic technique and divided the study groups accordingly. The significant variation in tissue stiffness from control, healthy endometrial tissue and the distinguishing of UFs and AM based on SR values proves the diagnostic potential of TVUS combined with SRE for the concomitant identification of these disorders, thus confirming hypotheses raised by literature data.

## 5. Conclusions

TVUS combined with SRE represents a potential reliable diagnostic imaging technique for the identification of concomitant UF and AM lesions, according to our study. A significant, obvious increase in tissular rigidity can be encountered in the case of both UF and AM in comparison with healthy patients, with the highest mean and max SR values being identified among patients with AM, the cut-off values obtained in the present study, which proved that tissular rigidity can successfully be used to differentiate UFs from AM. Future studies on wider populations are needed to confirm our findings, as well as research comparing TVUS and SRE with other imaging techniques, in order to establish the utility of the method in terms of diagnostic accuracy and cost-efficacy.

## Figures and Tables

**Table 1:** Baseline characteristics comparison between the two study groups.

Parameter		Study Group (n = 17)	Controls (n = 46)	*p* Value
Age (mean ± SD) ^a^		47.12 ± 4.10	37.8 ± 7.76	**<0.01**
BMI (mean ± SD) ^a^		25.31 ± 4.45	24.28 ± 3.25	0.39
Curettage history ^b^	Positive (%)	15.87	19.04	**0.01**, OR = 4.04 (95% CI: 1.20–12.97)
	Negative (%)	11.11	53.96	
C-section history ^b^	Positive (%)	4.76	19.04	0.48, OR = 0.60 (95% CI: 0.16–2.20)
	Negative (%)	22.22	53.96	

Legend: ^a^—*t* test with Welch’s correction was applied; ^b^—Chi square test was applied; BMI—body mass index; CI—confidence interval; SD—standard deviation.

**Table 2:** ROC analysis of mean and max SR values for differentiation between adenomyosis from controls.

Parameter	AUC (95% CI)	Cut Off	Sensitivity (95% CI)	Specificity (95% CI)	*p* Value
mean SR	1.00 (1-1)	>5.42	100% (79.61–100%)	100% (92.29–100%)	**<0.001**
max SR	1.00 (1-1)	>5.80	100% (79.61–100%)	100% (92.29–100%)	**<0.001**

Legend: SR—strain ratio; CI—confidence interval.

**Table 3:** ROC analysis of mean and max SR values for differentiation between leiomyoma and controls.

Parameter	AUC (95% CI)	Cut Off	Sensitivity (95% CI)	Specificity (95% CI)	*p* Value
mean SR	1.00 (1-1)	>2.85	100% (79.61–100%)	100% (92.29–100%)	**<0.001**
max SR	1.00 (1-1)	>3.30	100% (79.61–100%)	100% (92.29–100%)	**<0.001**

Legend: SR—strain ratio; CI—confidence interval.

## Data Availability

Data will be made available from the corresponding author upon reasonable request.
